# Buffalo Medical Association

**Published:** 1880-01

**Authors:** 


### Society Reports.

### BUFFALO MEDICAL ASSOCIATION.

*Stated Meeting, December 2, 1879.*

THE PRESIDENT, DR. LUCIEN HOWE, IN THE CHAIR.

An unusually large number of members were present, together with medical students and others specially invited. The paper was read by Honorable George W. Clinton whose subject was "Malpractice." (See original communications.)

When the speaker had concluded his paper, Dr. White moved that the thanks of the Association be tendered Judge Clinton for his able, eloquent and instructive address.

The subject of medical jurisprudence, said the Doctor, was one with which he had had very much to do. He had often been called upon to testify in cases of malpractice.

The medical expert, when upon the stand, is not expected to know everything. To questions involving an uncertainty, or touching the superiority of his own skill, it is best to say in reply that we do not know. We should not speak for ourselves, and always give good reasons for any statement made.

Thirty-five years ago he was sued for malpractice, when what is termed a "struck" or "select" jury was obtained. It was the only case of the kind ever occurring in this part of the State. He was sued for an imperfect cure of a fractured thigh. He proved, by the plaintiff's own witnesses, that it was an average cure, and that the directions were not obeyed. There were two trials, the jury disagreeing in both. When the case was finally presented to the select jury of more intelligent and unprejudiced men, and after hearing the testimony of such men as Willard Parker, Frank H. Hamilton, John Delamater, Prof. Ackley, of Cleveland, and many others, scarcely leaving their seats for deliberation, they immediately brought in a verdict for the defense, involving, however, great expense in defending an attack, the chief merit of which consisted of an assertion that the plaintiff was poor, with six children,

Till that time, suits for malpractice were of frequent occurrence, nearly all the leading practitioners having suits brought against them—the object of the plaintiff being to effect a compromise. In the case just narrated, this could have been done at a cost much less than that of a trial.

Prof. White was of the opinion that suits for malpractice were ordinarily the result of a thoughtless remark on the part of some rival practitioner. He always esteemed it his duty, as well as pleasure, to do what he could to defend a fellow-physician.

After dwelling at some length upon the unfairness of the present system, he called for the adoption of the motion.

Dr. Miner expressed his thanks to Judge Clinton for the lecture, and added that he had been almost convinced of the equity of our legal code. The "ruling of the Court" was not always the "ruling of the jury," which latter, unfortunately, was too often unjustly detrimental to the physician. If men are sufficiently intelligent to form an opinion on the question to be decided, they are, on that very account, disqualified to serve on a jury. Suits for malpractice are not infrequently "worked up" by unprincipled lawyers, who hope to benefit themselves and their clients financially, by robbing the physician of both money and reputation. Such legal adventurers are as numerous as are quacks in medicine. In closing, Dr. Miner again complimented the Judge upon the excellencies of the paper and hoped that the motion of Dr. White would be adopted.

Upon being put to vote, the resolution was adopted unanimously.

